# Synthesis of a Morpholino Nucleic Acid (MNA)-Uridine Phosphoramidite, and Exon Skipping Using MNA/2′-*O*-Methyl Mixmer Antisense Oligonucleotide

**DOI:** 10.3390/molecules21111582

**Published:** 2016-11-22

**Authors:** Suxiang Chen, Bao T. Le, Kamal Rahimizadeh, Khalil Shaikh, Narinder Mohal, Rakesh N. Veedu

**Affiliations:** 1 Centre for Comparative Genomics, Murdoch University, Perth 6150, Australia; S.Chen@murdoch.edu.au (S.C.); T.Le2@murdoch.edu.au (B.T.L.); rahimizadeh.kamal@gmail.com (K.R.); 2 Western Australian Neuroscience Research Institute, Perth 6150, Australia; 3 GMK Research Laboratories Pvt. Ltd., Mallapur, Hyderabad 500 076, India; khalil.shaikh@gmkresearch.com (K.S.); narinder.mohal@gmkresearch.com (N.M.)

**Keywords:** morpholino nucleotide, PMO, exon skipping, antisense oligonucleotide

## Abstract

In this study, we synthesised a morpholino nucleoside-uridine (MNA-U) phosphoramidite and evaluated the potential of a MNA-modified antisense oligonucleotide (AO) sequences to induce exon 23 skipping in *mdx* mouse myotubes in vitro towards extending the applicability of morpholino chemistry with other nucleotide monomers. We designed, synthesised, and compared exon skipping efficiencies of 20 mer MNA-modified 2′-*O*-methyl RNA mixmer AO on a phosphorothioate backbone (MNA/2′-OMePS) to the corresponding fully modified 2′-*O*-methyl RNA AO (2′-OMePS) as a control. Our results showed that the MNA/2′-OMePS efficiently induced exon 23 skipping. As expected, the 2′-OMePS AO control yielded efficient exon 23 skipping. Under the applied conditions, both the AOs showed minor products corresponding to exon 22/23 dual exon skipping in low yield. As these are very preliminary data, more detailed studies are necessary; however, based on the preliminary results, MNA nucleotides might be useful in constructing antisense oligonucleotides.

## 1. Introduction

Nucleic acid-based technologies [1] have attracted significant interest in recent years for targeting the molecular pathogenesis of various diseases. Antisense oligonucleotides (AOs), an important therapeutic molecule of this class, can efficiently regulate the expression of cellular RNAs by selectively cleaving, blocking, or repairing pathogenic messenger RNAs [1]. AOs bind to complementary target RNA sequences through hydrogen bonding. High affinity RNA targeting, better mis-match recognition, and a high degree of nuclease resistance are key factors in developing successful AO-based therapeutics. AOs composed of naturally occurring nucleotides show low target binding affinity and substantially poor resistance to nucleases, and consequently are unsuitable for therapeutic development. Chemically modified nucleotide analogues are generally incorporated into AOs to overcome these limitations and improve the pharmacokinetic profile of nucleic acid-based drugs. A number of chemically modified nucleotides have been developed in recent years. Some examples of the prominent chemistries utilised in AOs are 2′-*O*-methyl (2′-OMe) [2], 2′-*O*-methoxyethyl (2′-*O*-MOE) [3], phosphorodiamidate morpholino (PMO) [4], locked nucleic acid (LNA) [5,6], peptide nucleic acid (PNA) [7], tricyclo-DNA (tcDNA) [8], and unlocked nucleic acid (UNA) [9]. As the internucleotide linkages are vulnerable to nuclease cleavage, the non-bridging oxygen atom of the normal phosphate (as in DNA) backbone is often substituted with a sulphur atom to form a phosphorothioate (PS) linkage mainly to improve resistance [10], and phosphorothiolation helps to improve the in vivo circulation time of AOs by binding to serum albumin. Two AO candidates have so far been approved by the US FDA for clinical use: Vitravene, a 21 mer PS-modified AO against cytomegalovirus retinitis, and, more recently, Kynamro, another AO modified with 2′-*O*-MOE for the treatment of familial hypercholesterolemia [1].

**Figure 1:**
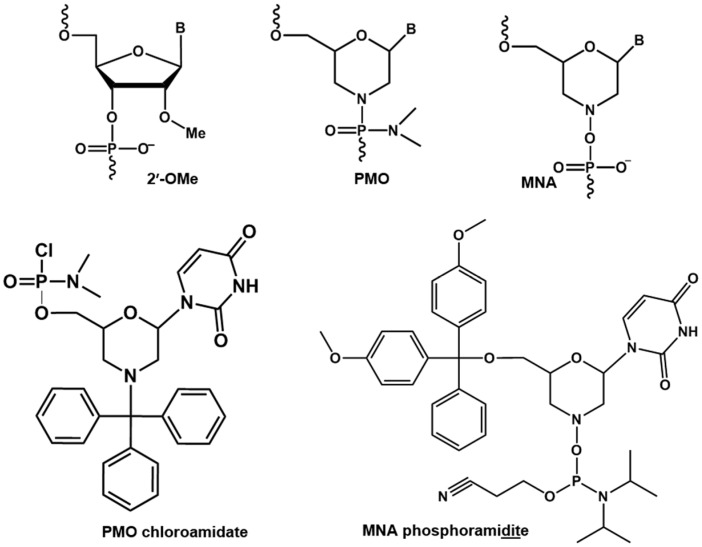
Structural representations of 2′-OMe, PMO, MNA monomers, and PMO chloroamidate and MNA phosphoramidite derivatives.

AOs can be used to correct the defects in pre-messenger RNAs (pre-mRNAs) by skipping or retaining a specific exon during the RNA splicing process within the nucleus [11]. Exon skipping has been explored as a treatment of Duchenne muscular dystrophy (DMD), a muscle wasting, invariably fatal genetic disease that mainly affecting boys and leads to death in early adulthood [12]. Individuals with DMD lack the protein dystrophin, which is required to strengthen and protect muscles from eccentric contraction [13]. There are 79 exons in the dystrophin transcript and DMD arises through mutations at various sites. AO-mediated exon skipping is currently the only approach that has shown promise in slowing disease progression. AOs act by annealing to the target pre-mRNA and by inducing skipping of exons adjacent to the disease-causing mutation. This leads to restoration of the open reading frame leading to the expression of a partially functional form of dystrophin. Two AO candidates, drisapersen (2′-OMePS chemistry, Figure 1) and eteplirsen (PMO chemistry, Figure 1), both of which exon 51, have been the subject of Phase III clinical trials [14,15,16,17]. US FDA rejected drisapersen mainly due to toxicity issues and its limited capacity to target muscle cells. Although an AO with PMO-chemistry was relatively non-toxic, it was necessary to administer it in very high doses (1 g per week), and AOs with PMO chemistry cannot currently be produced on the massive scale required to meet a potential global supply as the synthesis is challenging. In addition, PMO chemistry is not compatible with standard phosphoramidite chemistry to synthesise mixmer AOs with other modifications including LNA or 2′-OMe nucleotides to further improve the efficacy. To overcome this limitation, it is important to investigate the potential modified morpholino analogues. Towards this goal, herein, we report the synthesis of morpholino nucleic acid (MNA) uridine phosphoramidite (Figure 1) and the evaluation of MNA/2′-OMePS mixmer AO for inducing exon 23 skipping in *mdx* mouse myotubes.

## 2. Results and Discussion

### 2.1. Synthesis of MNA-Uridine Phosphoramidite

**Figure 2:**
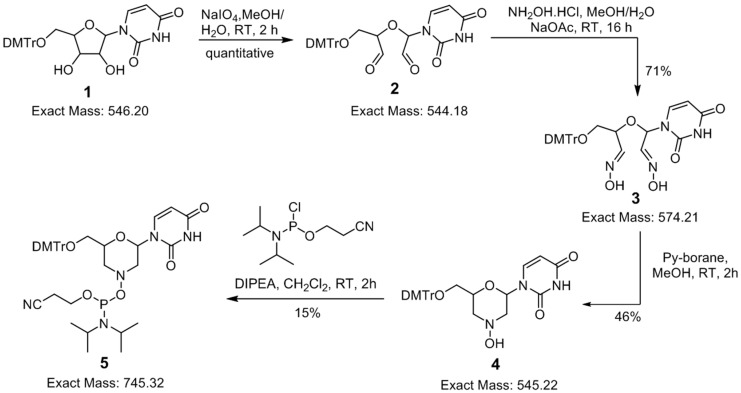
Synthesis of MNA-uridine phosphoramidite.

Inspired by the efficacy of PMO-based AO candidates to induce exon skipping in DMD and the recent clinical trials of PMO candidate eteplirsen, we envisaged the construction of a mixmer AO candidate with a morpholino ring containing a phosphodiester/phosphorothioate backbone (we referred to this as morpholino nucleic acid, MNA) via standard phosphoramidite chemistry. For this purpose, we synthesised morpholino-urdine (MNA-U) phosphoramidite, which is compatible with other nucleotide chemistries, to synthesise mixmer AOs using an oligonucleotide synthesiser. Towards the synthesis, the DMTr-protected uridine **1** [18] was first converted to dialdehyde **2** using classical sodium periodate-mediated oxidation in a quantitative yield, and the crude aldehyde **2** was further treated with hydroxyl amine hydrochloride to yield corresponding dioxime **3** in good yield (71%), which was purified by silica gel chromatography [19,20]. The treatment of dioxime **3** with pyridine-borane complex at room temperature gave DMTr-protected morpholino-uridine nucleoside **4** in 46% yields (slightly DMTr-group deprotection was observed under these conditions) [19]. Intermediate **4** was then treated with *N*,*N*-diisopropyl-cyanoethylphophorochloridite under standard phosphoramidite synthesis conditions (DIPEA, CH_2_Cl_2_, RT) to obtain corresponding phosphoramidite building block **5**, which was used further for the synthesis of oligonucleotides (Figure 2). The low phosphoramidite synthesis yield might be due to the susceptibility of phosphoramidite prepared from hydroxylamine (bond dissociation energy for the N–O bond is 55 kcal/mol, which is much lower than the C–O bond (85.5 kcal/mol). This may also explain the lower stability of phosphoramidite prepared from hydroxylamine compared with the standard phosphoramidites prepared from alcohols. 

### 2.2. Evaluation of Exon Skipping Using MNA-Modified 2′-OMePS AO

In our study, we used DMD as a model system to evaluate the potential of MNA-modified AOs. Two AO candidates were synthesised in one micromole scale, a fully modified 2′-OMe AO control (2′-OMePS, Table 1) and a MNA-modified 2′-OMePS (MNA/2′-OMePS, Table 1), which were designed to target *Dmd* exon 23, which carries a nonsense point mutation in the dystrophin gene pre-mRNA transcript. The MNA/2′-OMePS AO synthesised in this preliminary study had one MNA nucleotide at position 10. We performed the thermal stability experiments for both AOs using the complementary target RNA (5′-rAG GUA AGC CGA GGU UUG GCC-3′). Notably, the MNA/2′-OMePS was found to be less stable (55.4 °C) compared to the control 2′-OMePS AO (59.8 °C) (Table 1). To evaluate the efficacy of the AO sequences, *mdx* mice myoblasts were propagated as previously described [21]. Cells were plated at confluent density and allowed to differentiate for 1–3 days in a low serum medium prior to transfection. 2′-OMePS and MNA/2′-OMePS AOs were then transfected into differentiated myotubes in complex with cationic lipids (lipofectin, 1:1 *w*/*w* ratio) at 400 nM concentrations. Transfected cells were incubated for 24–48 h, and the total RNA was extracted using Trizol reagent according to the manufacturer’s instructions (Zymo Research, Orange, CA, USA), and RT-PCR was carried out as previously described by nested RT-PCR [22]. RT-PCR products were then analysed by 2% agarose gel electrophoresis. 

**Figure 3:**
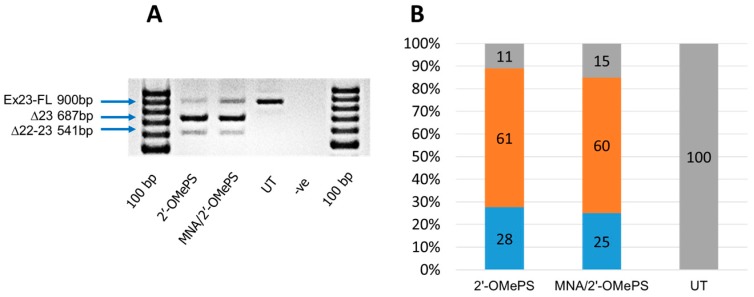
RT-PCR analysis (**A**) and densitometry analysis (**B**) of exon 23 skipping in cultured *mdx* myotubes. Orange colour: Percentage of exon-skipping product. Blue colour: Percentage of dual exon-22/23 skipped product. Grey colour: Percentage of full length product. UT: Untreated.

The results clearly showed that both MNA/2′-OMePS and the fully modified 2′-OMePS AOs induced efficient skipping of exon 23 (full length band at 900 bp) at a 400 nM concentration by yielding the 687 bp deletion product (Figure 3). Densitometry analysis of the gel images was performed to quantify the actual percentage of skipping (Figure 3B). Specifically, the 2′-OMePS AO achieved a higher percentage of exon skipping compared with the MNA/2′-OMePS mixmer AO (Figure 3A,B). However, under the applied conditions, the MNA/2′-OMePS mixmer AO showed the exon 23 deletion product of 687 bp in a similar yield at 400 nM (60%, Figure 3A,B), compared to the 2′-OMePS AO (61%, Figure 3A,B). Notably, both AOs showed an additional band at 541 bp, believed to be due to the undesired dual exon 22 and 23 skipping products (Figure 3A). This product was more evident in the case of the control 2′-OMePS AO (28%, Figure 3A,B) compared with the MNA/2′-OMePS mixmer AO (25%, Figure 3A,B). The efficacy of 2′-OMePS and the MNA/2′-OMePS AOs were analysed three times, and it was observed that the conventional 2′-OMePS was slightly better at inducing exon skipping (Figure S4, Supplementary information). Although these are our very preliminary data, more systematic studies are currently underway. To evaluate the cytotoxicity of the AOs, we then conducted a cell viability assay using WST-1 reagent (Sigma, St. Louis, MO, USA). Briefly, the cells were seeded and transfected with the AOs (400 nM) as described previously. After 24 h incubation, cells were combined with a WST-1 reagent at ratio 1:10 (*v*/*v*) per well and incubated for 2 h at 37 °C, 5% CO_2_, and the absorbance was measured at 450 nm by a plate reader. In general, there was no difference between cells treated with the 2′-OMePS AO and the MNA/2′-OMePS mixmer AO, which suggests that both AOs did not induce any cytotoxicity under the applied conditions (Figure S5, Supplementary Information).

## 3. Materials and Methods

### 3.1. Synthesis of MNA-Uridine Phosphoramidite

*3-(Bis(4-methoxyphenyl)(phenyl)methoxy)-2-(1-(2,4-dioxo-3,4-dihydropyrimidin-1(2H)-yl)-2-oxoethoxy)propanal* (**2**): To a stirred solution of compound **1** (41 g, 75.09 mmol) in MeOH/H_2_O (10:1, *v*/*v*, 450 mL), NaIO_4_ (24 g, 112.6 mmol) at room temperature (~28 °C) was added, and the reaction mixture was stirred for 2 h at the same temperature. The solvent was evaporated under vacuum, and the residue was taken in water (100 mL) and extracted with EtOAc (200 mL × 3). The combined organic phase was dried over Na_2_SO_4_ and evaporated under vacuum to yield compound **2** as white foam (40 g, quantitative). This compound **2** was used as such for the next step without purification. TLC: R*_f_* = 0.5 (10% MeOH in CH_2_Cl_2_). MS (ESI & APCI), (*m*/*z*) 543.1 (M − 1, −ve mode).

*(E/Z)-3-(Bis(4-methoxyphenyl)(phenyl)methoxy)-2-((E/Z)-1-(2,4-dioxo-3,4-dihydropyrimidin-1(2H)-yl)-2-(hydroxyimino)ethoxy)propanal oxime* (**3**): To an ice cooled solution of NH_2_OH-HCl (26 g, 36.76 mmol) in MeOH/H_2_O (1:9, *v*/*v*, 350 mL), NaOAc (30 g, 366 mmol) was slowly added. After 5 min, a solution of compound **2** (40 g) in MeOH (100 mL) was slowly added, and the reaction mixture was stirred overnight (~16 h) at room temperature. The solvent was evaporated under vacuum, and water was added to the resulting residue (100 mL) and extracted with EtOAc (200 mL × 3). The combined organic phase was dried over Na_2_SO_4_ and evaporated under vacuum. The crude residue was adsorbed on silica gel (neutralised with NEt_3_) and purified by column chromatography (silica gel, 230–400 mesh, 0%–10% MeOH in CH_2_Cl_2_) to yield compound **3** (27 g, white foam, 71% yield, mixture of regio and stereo isomers). TLC: R*_f_* = 0.3 (5% MeOH in CH_2_Cl_2_). ^1^H-NMR (DMSO-*d_6_*, 300 MHz): δ 11.70–11.21 (8s, 3H, D_2_O exchangable), 7.76–7.61 (m, 2H), 7.34–7.12 (m, 9H), 6.88–6.73 (m, 4H), 6.48, 6.21 (2t, 1H, *J* = 4.5 Hz & 4.5 Hz), 5.64 (t, 1H, *J* = 7.8 Hz), 4.28–4.10 (m, 1H), 3.73 (s, 6H), 3.25–3.03 (m, 2H); MS (ESI & APCI), (*m*/*z*) 573.3 (M − 1, −ve mode).

*1-(6-((Bis(4-methoxyphenyl)(phenyl)methoxy)methyl)-4-hydroxymorpholin-2yl)pyrimidine-2,4(1H,3H)-dione* (**4**): To an ice cooled solution of compound **3** (18 g, 31.35 mmol) in anhydrous MeOH (180 mL), BH_3_-pyridine complex (12 mL, ~8 M, 94.07 mmol) was added dropwise. After 5 min of stirring at ~10 °C, methanolic-HCl (6 mL, 4 M) was added slowly to adjust pH = 4.5–5. Then, the reaction mixture was stirred at room temperature for 2 h. The reaction was quenched with a solution of 10% aqueous NaOH saturated with NaCl, and the reaction mixture pH was adjusted to 7–8. Then, the product was extracted with EtOAc (200 mL × 3). The organic phase was dried over Na_2_SO_4_, evaporated under vacuum and purified by silica gel column chromatography (230–400 mesh, 10%–70% EtOAc in pet ether). The resulting main fraction was concentrated and re-purified by column chromatography using 1%–5% MeOH in CH_2_Cl_2_ to yield compound **4** (7.9 g, white foam, 46% yield). TLC: R*_f_* = 0.45 (3% MeOH in CH_2_Cl_2_). ^1^H-NMR (CDCl_3_, 300 MHz): δ 10.83 (s, 1H, D_2_O exchangeable), 7.44–7.39 (m, 3H), 7.30–7.18 (m, 7H), 6.80 (d, 4H, *J* = 8.7 Hz), 6.22 (d, 1H, *J* = 10.2 Hz), 5.77 (d, 1H, *J* = 7.8 Hz), 4.03 (m, 1H), 3.77 (s, 6H), 3.45–3.08 (m, 4H), 2.60 (m, 2H); MS (ESI & APCI), (*m*/*z*) 544.3 (M − 1, −ve mode); LCMS (Shimadzu LC-2020): 94.24% (purity) at 254 nm, *m*/*z* 544.3 (M − 1, −ve mode), (R_t_ = 9.78 min), (Column: CHEMSIL ODS C18 (150 × 4.6 mm), 5 µ, Mobile Phase A: 10 mM aqueous NH_4_OAc (pH = 7.0), B: MeCM, Gradient: (T/%B): 0/20, 8/90, 15/90; Flow:0.6 mL/min, Diluent: MeOH.

*2-((Bis(4-methoxyphenyl)(phenyl)methoxy)methyl)-6-(2,4-dioxo-3,4-dihydropyrimidin-1(2H)-yl)morpholino (2-cyanoethyl) diisopropylphosphoramidite* (**5**): To a solution of compound **4** (3.3 g, 6.05 mmol)) in anhydrous CH_2_Cl_2_ (35 mL), DIPEA (1.6 mL, 9.07 mmol) at ice bath temperature (0–5 °C) was added. After 5 min of stirring, 2-cyanoethyl-diisopropylchlorophosphoramidite (1.72 g, 7.26 mmol) was added over a period of 15 min at the same temperature. The reaction mixture was slowly warmed to room temperature, and additional 2-cyanoethyl-diisopropylchlorophosphoramidite (855 mg, 3.61 mmol) was added after 1 h of stirring over a period of 15 min and stirred for an additional 1 h. The reaction mixture was quenched with 10% aqueous NaHCO_3_ solution (25 mL) and extracted with CH_2_Cl_2_ (50 mL × 3). The combined organic phase was dried over Na_2_SO_4_ and evaporated under vacuum, and the residue was purified by silica gel column chromatography (neutralised with Et_3_N, 230–400 mesh, 0.5% MeOH in CH_2_Cl_2_/acetone (95:5, *v*/*v*) with 0.1% Et_3_N to 3% MeOH in CH_2_Cl_2_/acetone (85:15, *v*/*v*) with 0.1% Et_3_N) to yield target compound **5** (700 mg, white foam, 15% yield). TLC: R*_f_* = 0.45 (3% MeOH in CH_2_Cl_2_/acetone, 85:15, *v*/*v*, with 0.1% Et_3_N). ^1^H-NMR (CDCl_3_, 400 MHz): δ 7.44–7.38 (m, 3H), 7.31–7.18 (m, 8H), 6.83–6.80 (d, 4H, *J* = 8.4 Hz), 5.75–5.62 (m, 2H), 4.26–3.94 (m, 3H), 3.79 (s, 6H), 3.64–3.27 (m, 4H), 3.11–3.07 (m, 1H), 2.81–2.66 (m, 3H), 2.37 (t, 1H, *J* = 8.4 Hz), 2.17–1.98 (m, 1H), 1.36–1.25 (m, 12H) (mixture of diastereomers); ^31^P-NMR (CDCl_3_, 161 MHz): δ 15.93, 15.22 (mixture of diastereomers); MS (ESI & APCI), (*m*/*z*) 744.6 (M − 1, −ve mode); LCMS (Shimadzu LC-2020, Kyoto, Japan): 94.80% (purity) at 254 nm, *m*/*z* 744.6 (M − 1, −ve mode), (R_t_ = 11.33 min), (Column: CHEMSIL ODS C18 (100 × 4.6 mm), 3 µ, Mobile Phase A: 10 mM aqueous NH_4_OAc (pH = 7.0), B: MeCN, Gradient: (T/%B): 0.01/5, 10/90, 16/90; 0.6 mL/min, Diluent: MeCN. HPLC (Shimadzu LC-2010CHT): 94.75% (purity) at 254 nm, (R_t_ = 17.64 min), (Column: SYNCRONIS C18 (250 × 4.6 mm), 5 µ, Mobile Phase A: 10 mM aqueous NH_4_OAc (pH = 7.0), B: MeCN, Gradient: (T/%B): 0.01/5, 15/90, 20/90; Flow: 1.0 mL/min, Diluent: MeCN.

### 3.2. Synthesis of Chemically Modified Antisense Oligonucleotides (AOs)

#### 3.2.1. Oligonucleotide Synthesis

All AOs (Table 1) were prepared in-house on an Expedite 8909 DNA synthesiser via standard phosphoramidite chemistry in a 1 µmol scale (Activator: 1*H*-tetrazole, Sulfurising agent: 0.02 M Xanthane hydride in Pyridine/Acetonitrile (*v*/*v* = 1:4). For MNA-uridine phosphoramidite, the coupling time was prolonged to 15–20 min, while the normal coupling time for 2′-OMe phosphoramidite was only 6 min. Synthesised oligonucleotides were deprotected and cleaved from the solid support by treatment with NH_4_OH at 55 °C overnight. The crude oligonucleotides were then purified by preparative polyacrylamide gel-electrophoresis (Figures S1–S3; Supplementary Information), desalted and verified by MALDI-ToF MS analysis. 

#### 3.2.2. Melting Temperature Study of the Antisense Oligonucleotides (AOs)

Two antisense oligonucleotides: control 2′-OMePS and MNA/2′-OMePS were prepared at a 2 µM concentration in a buffer containing 10 mM NaCl and 0.01 mM EDTA, adjusted to pH 7.0 by 10 mM sodium phosphate buffer. The AOs were then mixed with the complementary RNA sequence (2 µM) at equal volume and denatured at 95 °C for 10 min followed by slow cooling to room temperature and loaded onto 1 mm path-length quartz cuvettes. The *T*_m_ was analysed on Shimadzu UV-1800 instrument with the temperature controller with the range of 20–90 °C with a ramp rate of 1.0 °C/ min. *T*_m_ values were calculated by the first derivative.

### 3.3. Evaluation of Exon Skipping Using the MNA-Modified 2′-OMePS AO

#### 3.3.1. Cell Culture and Transfection

Immortalised *mdx* myoblasts (H2K cells, provided by Prof. Sue Fletcher and Prof. Steve Wilton’s laboratory, Murdoch University, Perth, Australia) were cultured as described previously [21,22]. Briefly, when 60%–80% confluent, primary *mdx* myoblast cultures were treated with trypsin (Life Technologies) and seeded at a density of 2 × 10^4^ cells/well into 24 well plates. The plate was pre-treated with 50 μg/mL poly-d-lysine (Sigma, St. Louis, MO, USA) and 100 μg/mL Matrigel (Corning, New York, NY, USA). Cultures were induced to differentiate into myotubes in Dulbecco’s Modified Eagle Medium (DMEM) containing 5% horse serum by incubation at 37 °C, 5% CO_2_ for 48 h. Antisense oligonucleotides were complexed with Lipofectin (Life Technologies, Carlsbad, CA, USA) at the ratio of 2:1 (lipofectin:AO) and used in a final transfection volume of 500 μL/well in a 24-well plate as per the manufacturer’s instructions, except that the solution was not removed after 3 h. 

#### 3.3.2. RNA Extraction and Reverse Transcription-Polymerase Chain Reaction (RT-PCR)

RNA was extracted from transfected cells using Direct-zol™ RNA MiniPrep Plus with TRI Reagent^®^ (Zymo Research) as per the manufacturer’s instructions. The dystrophin transcripts were then analysed by nested RT-PCR across exons 20–26 as described previously [22]. PCR products were separated on 2% agarose gels in Tris–acetate–EDTA buffer, and the images were captured on a Fusion Fx gel documentation system (Vilber Lourmat, Marne-la-Vallee, France). Densitometry was performed by Image J software (National Institute of Health, Bethesda, MD, USA).

#### 3.3.3. In Vitro Evaluation of Cell Viability

Cells were seeded and transfected with AOs as described above. After 24 h, cell viability was measured with a WST-1 assay kit (Sigma). Briefly, the WST-1 solution was added at ratio 1:10 (*v*/*v*) per well and incubated for 2 h at 37 °C, 5% CO_2_. The absorbance was measured on a microplate reader (FLUOstar Omega, BMG Labtech, Germany) at 450 nm.

## 4. Conclusions

To summarise, we synthesised a morpholino nucleoside-uridine phosphoramidite and a 20 mer morpholino nucleic acid (MNA)-modified 2′-OMe mixmer AO sequence was synthesised to investigate its potential in inducing exon 23 skipping in *Dmd* gene transcript in mouse myotubes in vitro. Our results showed that the MNA-modified 2′-OMePS AO efficiently induced exon 23 skipping in parallel to the fully modified 2′-OMePS AO control. Although these are only our preliminary studies, and more detailed investigations are essential for further verifications, based on the results, we believe that MNA-modified AOs could be used in constructing therapeutic antisense oligonucleotides for exon skipping and other antisense mechanisms. 

## Figures and Tables

**Table 1:** Antisense oligonucleotide sequences used in this study.

AO Names	Sequence, 5′→3′ Direction	*T*_m_, °C
2′-OMePS	GGCCAAACCUCGGCUUACCU	59.8
MNA/2′-OMePS	GGCCAAACC**U^M^**CGGCUUACCU	55.4

MNA nucleotide is represented in bold underlined with a superscript ‘M’.

## Supplementary Materials

The following are available online at http://www.mdpi.com/1420-3049/21/11/1582/s1.

## Abbreviations:

The following abbreviations are used in this manuscript:

2′OMe	2′-*O*-methyl
AO	antisense oligonucleotides
DMEM	Dulbecco’s Modified Eagle Medium
DMD	Duchenne muscular dystrophy
DNA	deoxyribonucleic acid
EDTA	ethylenediaminetetraacetic acid
FDA	Food and Drug Administration
HPLC	high-performance liquid chromatography
LCMS	liquid chromatography-mass spectrometry
LNA	locked nucleic acid
MNA	morpholino nucleic acid
MS	mass spectrometry
NMR	nuclear magnetic resonance
PNA	peptide nucleic acid
PMO	phosphorodiamidate morpholino
PS	phosphorothioate
RNA	ribonucleic acid
RT-PCR	reverse-transcriptase polymerase chain reaction
UNA	unlocked nucleic acid
