# Supplementary material for: Synthesis of a Morpholino Nucleic Acid (MNA)-Uridine Phosphoramidite, and Exon Skipping Using MNA/2′-O-Methyl Mixmer Antisense Oligonucleotide
Source: Molecules. 2016 Nov 22;21(11):1582. doi: 10.3390/molecules21111582 (PMC6274534; doi:10.3390/molecules21111582)
Supplement: Supplementary file 1 [file molecules-21-01582-s001.pdf]

Supplementary Materials: Synthesis of Morpholino Nucleic Acid (MNA)-Uridine Phosphoramidite, and Exon Skipping Using MNA/2′-*O*-Methyl Mixmer Antisense Oligonucleotides

Suxiang Chen, Bao T. Le, Kamal Rahimizadeh, Khalil Shaikh, Narinder Mohal and Rakesh N. Veedu

1. Purity of the Synthesized Antisense Oligonucleotides

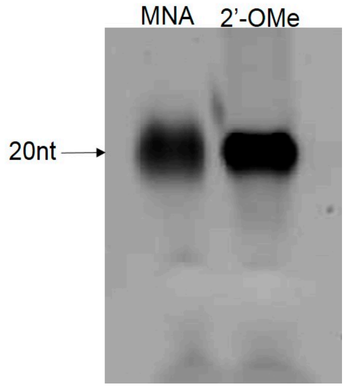

Figure S1. Analysis of the purity of the synthesized AOs by 15% denaturing polyacrylamide gel electrophoresis.

2. MALDI-ToF MS Analysis of Antisense Oligonucleotides

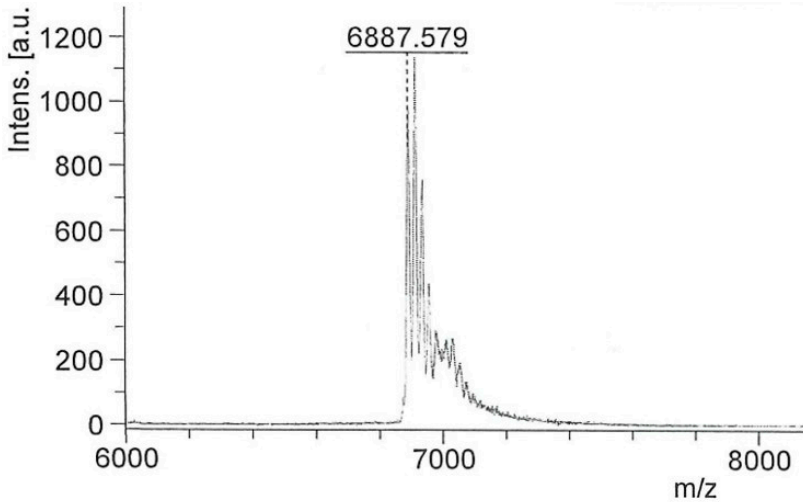

Figure S2. MALDI-ToF MS analysis of 2′-OMePS AO. Calculated: 6887.5 Da; Found: 6887.579 Da.

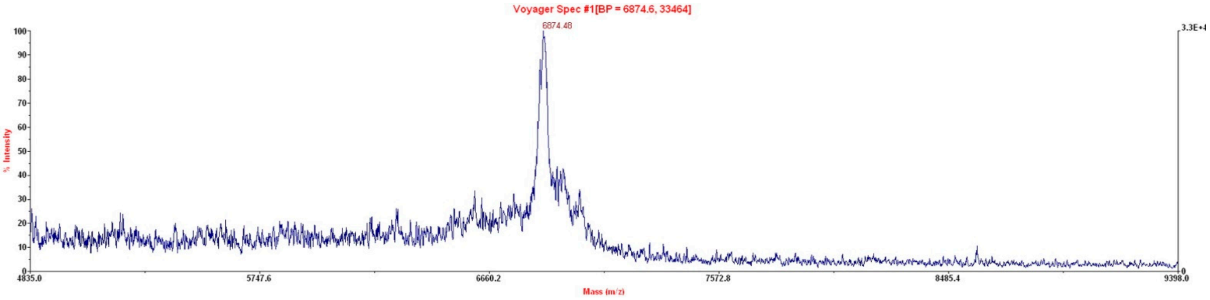

Figure S3. MALDI-ToF MS analysis of MNA/2′-OMePS AO. Calculated: 6872.5 Da; Found: 6874.48 Da.

3. RT-PCR and Densitometry Analysis Data

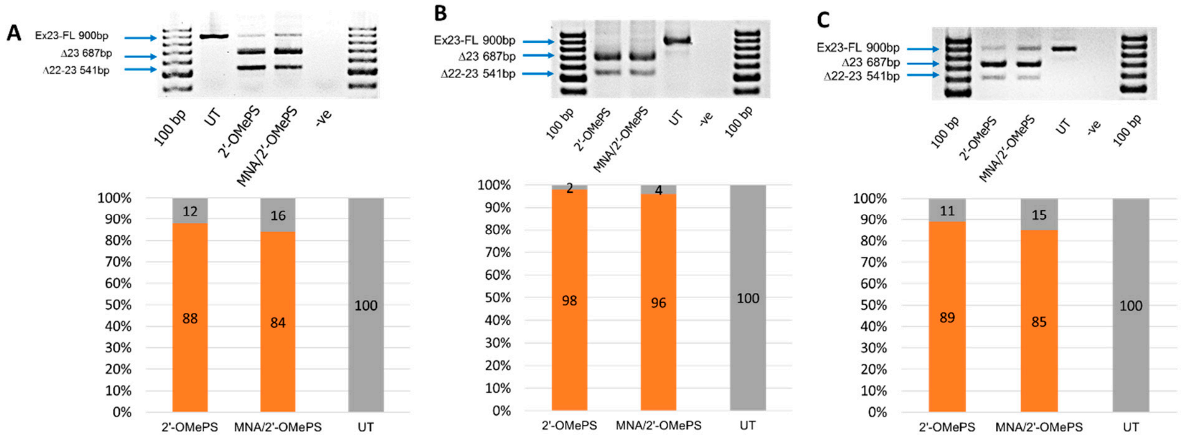

Figure S4. RT-PCR and densitometry analysis of exon 23 skipping in cultured *mdx* myotubes. A, B, C: RT-PCR results and densitometry data shown in triplicates. Orange: Percentage of exon-skipping products; Grey: Percentage of full length product; UT: Untreated.

4. In Vitro Evaluation of Cell Viability

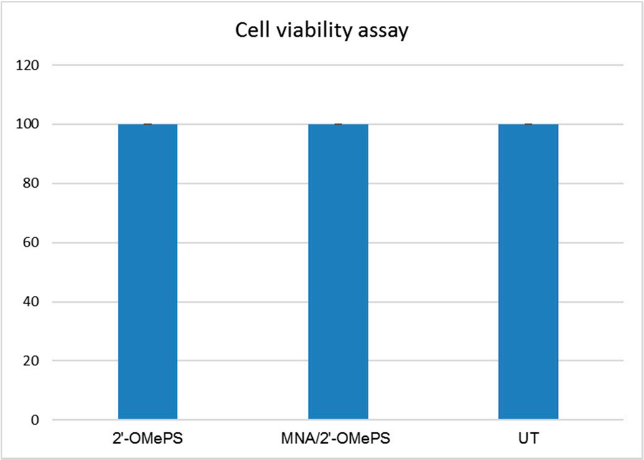

Figure S5. Cell viability assay after 24 h of transfection. UT: Untreated.
